# Case Report: A large right atrial hemangioma: multimodal imaging and postoperative follow-up

**DOI:** 10.3389/fradi.2026.1821324

**Published:** 2026-07-30

**Authors:** Tianhe Ye, Cong Liu

**Affiliations:** 1 Department of Radiology, Union Hospital, Tongji Medical College, Huazhong University of Science and Technology, Wuhan, China; 2 Hubei Province Key Laboratory of Molecular Imaging, Wuhan, China; 3 Department of Ultrasound Medicine, Union Hospital, Tongji Medical College, Huazhong University of Science and Technology, Wuhan, China

**Keywords:** cardiac neoplasms, case reports, multimodal imaging, postoperative follow-up, right atrial hemangioma

## Abstract

Primary cardiac tumors are extremely rare, and hemangiomas represent only a small subset, with right atrial involvement being particularly uncommon, posing diagnostic challenges due to non-specific symptoms and overlapping imaging features. We present a 55-year-old male with an incidentally discovered right atrial mass initially suspected to be a myxoma. Multimodal imaging with transthoracic echocardiography (TTE) and cardiac magnetic resonance (CMR) was performed: TTE identified a large mobile mass causing partial tricuspid obstruction, while CMR with quantitative diffusion-weighted imaging/apparent diffusion coefficient (DWI/ADC) provided detailed tissue characterization and anatomical mapping, revealing typical features of a hemangioma. Surgical resection was performed via median sternotomy under cardiopulmonary bypass. The mass, arising from the right atrial posterior wall and interatrial septum, was completely excised, and the atrial defect was repaired with a pericardial patch. Histopathology and immunohistochemistry confirmed a benign capillary hemangioma. Postoperative echocardiography demonstrated reduced right atrial diameter, resolution of obstruction, and mild stable tricuspid regurgitation, with no major complications. Combined TTE and CMR multimodal imaging is crucial for accurate preoperative diagnosis and surgical planning of right atrial hemangiomas. Complete surgical resection achieves favorable outcomes, and long-term multimodal imaging follow-up is recommended to exclude recurrence.

## 1 Introduction

Primary cardiac tumors are rare in the general population, with an incidence of approximately 0.001%–0.03%. Hemangiomas represent less than 2% of all primary lesions, and right atrial involvement is seen in fewer than 10% of cardiac hemangiomas (1). The clinical diagnosis of right atrial hemangioma faces two significant challenges. First, its clinical presentation is highly non-specific: most lesions are detected incidentally during imaging for unrelated conditions, while large space-occupying masses may cause severe complications including heart failure, arrhythmias, or systemic embolization. Second, its imaging features overlap with more common cardiac masses such as atrial myxoma, lipoma, and metastatic tumors (2). Imaging plays a pivotal role in distinguishing benign from malignant cardiac masses: transthoracic echocardiography (TTE) serves as the first-line modality for initial detection of cardiac masses, as it offers the advantages of accessibility and real-time assessment of mass mobility and valvular obstruction (3); cardiac magnetic resonance (CMR) complements TTE effectively by providing superior soft-tissue contrast, multiplanar anatomical detail, and comprehensive tissue characterization through sequences such as T1/T2 weighting, diffusion-weighted imaging (DWI), and late gadolinium enhancement (LGE) (4, 5). In this report, we present a case of a large right atrial hemangioma that was diagnosed using multimodal imaging (TTE combined with CMR), with confirmation through surgical intervention and pathological examination, and this case aims to highlight the complementary value of these two imaging techniques in the management of rare cardiac tumors.

## 2 Case description

### 2.1 Clinical presentation

A 55-year-old male was admitted to our hospital for further evaluation of a right atrial mass. His past medical history included a diagnosis of hypertensive intracerebral hemorrhage at a local hospital 1.5 years prior, during which a transthoracic echocardiography (TTE) was performed and revealed an abnormal echo in the right atrium, which was preliminarily diagnosed as an atrial myxoma without any subsequent intervention. At the time of admission, the patient had no symptoms of chest tightness, chest pain, dyspnea, or syncope, and he reported normal appetite, stable weight, and no experience of fatigue. Since the intracerebral hemorrhage 1.5 years ago, the patient had been experiencing right-sided hemiplegia and aphasia. Physical examination showed that the muscle strength of his right upper limb was Grade 3 according to the Medical Research Council scale. The patient had no family history of cardiac tumors or hereditary cardiovascular diseases, and no specific intervention was performed for the right atrial mass after its initial detection. The detailed clinical timeline of the patient's diagnosis and treatment is summarized in Table 1.

**Table 1:** Clinical timeline of the patient.

Time point	Key clinical event
1.5 years before admission	Diagnosed with hypertensive intracerebral hemorrhage; right atrial abnormal echo incidentally found on TTE, preliminarily diagnosed as atrial myxoma; no intervention performed
Admission	Admitted for further evaluation of the right atrial mass; asymptomatic for chest discomfort, dyspnea or syncope; physical examination showing right hemiplegia, aphasia, and right upper limb muscle strength Grade 3
Hospital day 2	Laboratory tests completed; TTE re-examination confirming a 7.5 × 3.1 cm right atrial mass with partial tricuspid obstruction
Hospital day 5	CMR examination performed, showing features consistent with benign hemangioma, with quantitative ADC value of 1.58 × 10⁻^3^ mm^2^/s
Hospital day 8	Complete surgical resection of the right atrial mass via median sternotomy under cardiopulmonary bypass
Postoperative week 1	TTE showing small pericardial effusion (maximum 10 mm); no arrhythmia, pericardial hemorrhage or other major complications
Postoperative week 2	TTE showing reduced right atrial transverse diameter (4.1 cm) and minimal residual fibrous scar tissue; high-sensitivity troponin I returned to normal (12.3 ng/L)

### 2.2 Laboratory findings

Laboratory and biochemical tests were conducted upon admission, and the results were as follows: complete blood count showed a white blood cell count of 3.87 × 10⁹/L (reference range: 4.0–10.0 × 10⁹/L), a hemoglobin level of 132 g/L (reference range: 120–160 g/L), and a platelet count of 81 × 10⁹/L (reference range: 125–350 × 10⁹/L); coagulation function tests showed a prothrombin time of 13 s (reference range: 11.0–14.2 s) and an activated partial thromboplastin time of 36.0 s (reference range: 28.0–43.5 s); cardiac biomarkers showed a high-sensitivity troponin I level of 91.7 ng/L (reference range: <26.2 ng/L) and an N-terminal pro-B-type natriuretic peptide (NT-proBNP) level of 77.8 pg/mL (reference range: <125 pg/mL); liver and renal function tests showed an aspartate aminotransferase level of 19 U/L (reference range: 8–40 U/L), an alanine aminotransferase level of 31 U/L (reference range: 5–40 U/L), and a serum creatinine level of 77.2 μmol/L (reference range: 44–133 μmol/L).

### 2.3 Transthoracic echocardiography (TTE) findings

**Figure 1:**
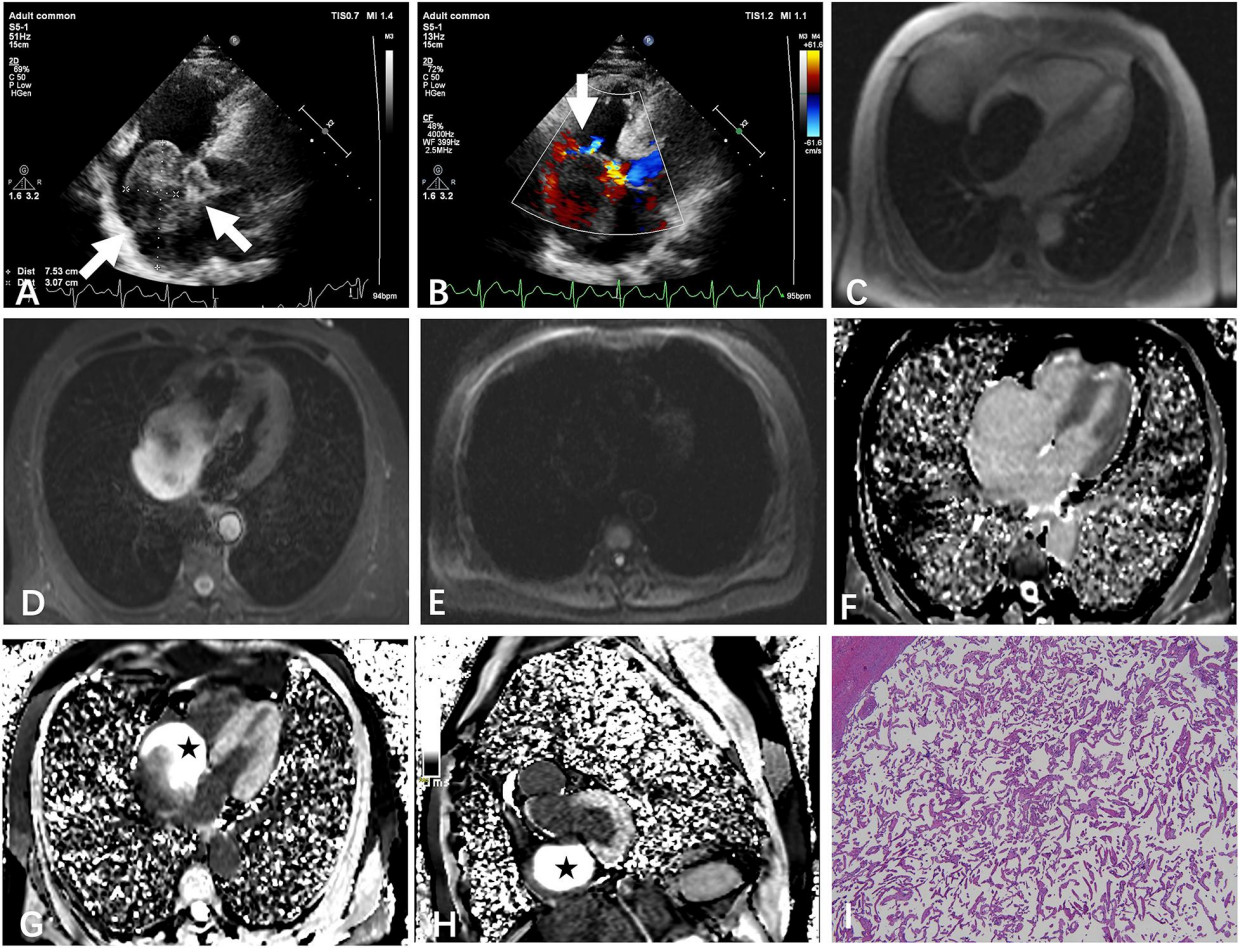
We present a 55-year-old male with a 1.5-year history of hypertensive intracerebral hemorrhage. Prior transthoracic echocardiography (TTE) incidentally found a right atrial (RA) abnormal echo, suspected as atrial myxoma without intervention. On admission, he was asymptomatic but with elevated high-sensitivity troponin I (91.7 ng/L), and reduced platelets (81 × 10⁹/L). Admission TTE showed a 7.5 cm × 3.1 cm hypoechoic RA mass, with partial ill-defined borders with the RA posterior wall and interatrial septum [panel **(A)**, white arrows], high mobility causing tricuspid obstruction; color Doppler detected tortuous internal blood flow [panel **(B)**, white arrow]. Cardiac magnetic resonance (CMR) (3.0 T Siemens Aera) confirmed the mass's mobility and partial tricuspid obstruction, and revealed hypointense on T1-weighted imaging (T1WI) [panel **(C)**] and hyperintense on fat-saturated T2-weighted imaging (T2WI) [panel **(D)**], with no diffusion restriction [apparent diffusion coefficient [ADC] 1.58 × 10⁻^3^ mm^2^/s, panel **(E)** [diffusion-weighted imaging, DWI] and F [ADC]] and heterogeneous late enhancement [size 7.7 cm × 5.1 cm × 6.9 cm, panel **(G)** [axial view,«] and H [oblique sagittal view,«]]. Surgical resection found a reddish-purple mass; pathology confirmed capillary hemangioma [hematoxylin-eosin (HE) staining showed capillaries lined with flattened endothelial cells; CD34+/CD31+/ERG+/D2–40-, panel **(I)**]. Postoperatively, 1-week TTE showed small pericardial effusion; 2-week TTE showed reduced RA diameter (4.1 cm × 4.7 cm) and normal troponin. Primary cardiac tumors are rare (0.001%–0.03%), with hemangiomas accounting for 2%–3% and RA involvement < 10%. Multimodal imaging (TTE for function, CMR for tissue characterization) is essential for diagnosis and safe surgery.

Transthoracic echocardiography (TTE) was performed using a Philips EPIQ 7C ultrasound system, with standard views including parasternal long-axis, parasternal short-axis, apical four-chamber, and subcostal views. The key findings of TTE included a right atrial transverse diameter of 4.7 cm (normal reference: <4.0 cm), a 7.5 × 3.1 cm hypoechoic mass located in the right atrial cavity and attached to the posterosuperior wall of the right atrium and the interatrial septum (with partial ill-defined borders), high mobility of the mass that swung with the cardiac cycle (moving toward the right atrial appendage during systole and prolapsing into the tricuspid orifice during diastole, causing partial obstruction) (Figure 1A), tortuous blood flow signals within the mass detected by color Doppler flow imaging (suggesting vascularization), a left ventricular ejection fraction (LVEF) of 60% (normal reference: >50%), and mild tricuspid regurgitation (Figure 1B).

### 2.4 Cardiac magnetic resonance (CMR) findings

Cardiac magnetic resonance (CMR) was performed on a 3.0 T Siemens Aera MRI scanner with a cardiac phased-array coil, and the sequences and parameters used were as follows: axial/coronal/sagittal T1-weighted imaging (T1WI) with a repetition time (TR) of 600 ms, an echo time (TE) of 15 ms, and a slice thickness of 5 mm; axial fat-saturated T2-weighted imaging (T2WI) with a TR of 3,000 ms, a TE of 80 ms, and a slice thickness of 5 mm; cine MRI with short-axis and four-chamber views, a TR of 30 ms, a TE of 1.5 ms, and a slice thickness of 5 mm; diffusion-weighted imaging (DWI) and apparent diffusion coefficient (ADC) mapping with b-values of 0 and 1,000 s/mm^2^, a TR of 4,000 ms, and a TE of 80 ms; late gadolinium enhancement (LGE) imaging performed 10 min after intravenous administration of gadobutrol (0.1 mmol/kg) with a TR of 800 ms, a TE of 20 ms, and a slice thickness of 5 mm. The key findings of CMR included the mass showing hypointense signal relative to myocardium on T1WI (Figure 1C)and hyperintense signal on fat-saturated T2WI (Figure 1D), cine MRI confirming the mass's mobility and partial tricuspid obstruction (consistent with TTE findings), no diffusion restriction of the mass with an ADC value of 1.58 × 10⁻^3^ mm^2^/s (a value normal for benign vascular lesions) (Figures 1E,F), scattered punctate hyperintensities in the left ventricular subendocardium on LGE imaging (consistent with minor myocardial injury resulting from right heart strain), heterogeneous enhancement of the right atrial mass after contrast administration (Figures 1G,H)—a typical feature of hemangiomas due to the variable vascular density within the lesion—a measured mass size of 7.7 × 5.1 × 6.9 cm (larger than the size measured by TTE, reflecting the multiplanar accuracy of CMR), and partial ill-defined borders between the mass and the right atrial posterior wall as well as the interatrial septum.

### 2.5 Surgical treatment

The patient underwent surgical resection of the right atrial mass under general anesthesia. First, a median sternotomy was performed, and cardiopulmonary bypass was established via aortic and bicaval cannulation. Then, the right atrium was opened with a longitudinal incision, and a soft, reddish-purple mass attached to the right atrial posterior wall and the interatrial septum was identified intraoperatively (consistent with the findings of CMR). Next, the mass was carefully dissected to avoid injury to the tricuspid valve and the coronary sinus; intraoperative inspection revealed that the base of the mass was fused with the interatrial septum, and the mass showed infiltrative growth that extended across the atrial groove to the left atrial posterior wall near the orifice of the right pulmonary vein. Finally, complete gross resection of the mass was achieved, and the defect in the right atrial wall was repaired using a bovine pericardial patch.

### 2.6 Pathological findings

Pathological examination was conducted on the resected specimen, which was a cystic-walled tissue measuring 5 × 3 × 1.5 cm; the cut surface of the specimen showed a grayish white to brownish appearance, a soft consistency, and no evidence of fibrosis or calcification. Hematoxylin-eosin (HE) staining showed a well-circumscribed lesion composed of numerous small capillaries, each lined by flattened and uniform endothelial cells, with abundant red blood cells present in the lumens of the capillaries (Figure 1I); no cellular atypia, mitotic figures, or necrosis was observed in the lesion, which was consistent with the diagnosis of a benign capillary hemangioma. Immunohistochemical staining was performed, and the results showed positivity for endothelial markers: strong membranous staining for CD34, cytoplasmic staining for CD31, and nuclear staining for ERG; the staining result for D2–40 (a lymphatic endothelial marker) was negative.

### 2.7 Postoperative follow-up and clinical outcomes

Postoperative follow-up was conducted for the patient. At 1 week postoperatively, transthoracic echocardiography was performed, and the results showed a small pericardial effusion (with a maximum thickness of 10 mm) and no pericardial hemorrhage; no arrhythmias were documented during this follow-up period. At 2 weeks postoperatively, repeat transthoracic echocardiography was performed. The results showed a right atrial transverse diameter of 4.1 cm (improved compared with the preoperative diameter of 4.7 cm), minimal residual hyperechoic tissue in the right atrium (considered as postoperative fibrous scar rather than residual tumor), and persistent mild tricuspid regurgitation (no progression compared with the preoperative condition). Detailed postoperative echocardiographic features are illustrated in the Supplementary Figure. Additionally, the level of high-sensitivity troponin I, a cardiac biomarker, returned to normal (12.3 ng/L) at 2 weeks postoperatively.

A standardized long-term surveillance plan has been scheduled for this patient to rule out tumor recurrence, given the infiltrative growth potential of cardiac hemangiomas. Follow-up arrangements include TTE at 6 and 12 months postoperatively, and CMR at 12 months for comprehensive tissue and anatomical evaluation. Annual clinical and echocardiographic follow-up will be continued thereafter.

## 3 Discussion

### 3.1 Clinical features

Right atrial hemangiomas represent an exceptionally rare subset of benign primary cardiac neoplasms, with fewer than 50 cases described in the English-language literature to date, which poses substantial diagnostic challenges for clinicians (1). The clinical course of these lesions is typically indolent: most cases remain asymptomatic and are discovered incidentally during cardiac imaging performed for non-cardiac indications, while large space-occupying lesions may lead to adverse clinical events via mechanical disruption, including tricuspid valve obstruction, right heart failure, and systemic thromboembolism (1, 6). The patient in this report presented with an incidentally detected large right atrial hemangioma, with a prior history of hypertensive intracerebral hemorrhage.

Regarding the causal relationship between right atrial hemangioma and intracerebral hemorrhage in this case, although right atrial hemangioma as an intracardiac mass could theoretically cause paradoxical embolism via intracardiac shunts, current evidence provides limited support for a direct link between this mechanism and parenchymal brain hemorrhage (7, 8). Paradoxical embolism, typically mediated by patent foramen ovale (PFO), is widely recognized to be mainly associated with ischemic stroke and serves as a common cause of cryptogenic stroke, while its causal association with intracerebral hemorrhage remains unestablished (9); the absence of a detectable intracardiac shunt on multimodal imaging further rules out paradoxical embolism as the underlying etiology. Additionally, as dedicated cerebrovascular imaging was not performed at the time of the hemorrhagic event, a concurrent intracranial hemangioma as the primary bleeding source cannot be definitively excluded from differential diagnosis. Given that hypertension is the most common cause of non-traumatic intracerebral hemorrhage and hypertension-related small vessel disease carries a significantly higher risk of bleeding than intracranial hemangiomas (10, 11), the intracerebral hemorrhage in this case is unlikely to result from paradoxical embolism secondary to right atrial hemangioma, and common etiologies such as hypertension-related small vessel disease should be prioritized in clinical evaluation.

### 3.2 Differential diagnosis

Right atrial masses often exhibit overlapping imaging features, which makes differential diagnosis a critical step in guiding clinical management decisions. Atrial myxoma is the most common differential diagnosis for right atrial masses. On TTE, it typically presents as a hyperechoic, polypoid lesion (12). Most atrial myxomas are attached to the interatrial septum at the fossa ovalis, and left atrial involvement is far more frequent than right atrial involvement (13, 14). On cardiac magnetic resonance (CMR), atrial myxoma shows isointense signal on T1-weighted imaging (T1WI) and hyperintense signal on T2-weighted imaging (T2WI), and it exhibits homogeneous enhancement after the administration of contrast agent (15, 16); histopathologically, atrial myxoma is characterized by the presence of myxoma cells, a feature that clearly distinguishes it from hemangioma (17).

Cardiac lipoma is another type of benign right atrial mass that can be differentiated from hemangioma using multimodal imaging. On TTE, cardiac lipoma appears as a hyperechoic lesion; CMR is highly specific for the diagnosis of cardiac lipoma due to the unique fat signal characteristics of the tumor—specifically, cardiac lipoma shows hypointense signal on both T1WI and T2WI when fat saturation sequences are applied, and it lacks vascularization [no blood flow signals are detected on color Doppler flow imaging (CDFI)], which is in clear contrast to the vascularized nature of hemangioma (18, 19).

Metastatic cardiac tumors must also be excluded during the differential diagnosis of right atrial masses, especially in patients with a history of extra-cardiac malignancy. On TTE, metastatic cardiac tumors typically have irregular borders, and they exhibit invasive growth patterns that may involve multiple cardiac chambers (20). On CMR, the signal characteristics of metastatic cardiac tumors are variable, with inconsistent signal intensities on T1WI and T2WI and heterogeneous late gadolinium enhancement (LGE) (20, 21); histopathological examination of metastatic cardiac tumors confirms the presence of malignant features such as cellular atypia, mitotic figures, or invasive growth, which are absent in benign hemangiomas (22).

In contrast, the right atrial hemangioma in this case showed distinct imaging and pathological features that supported its diagnosis: on TTE, the mass appeared as a hypoechoic lesion with internal blood flow signals (consistent with vascularization) and high mobility; on CMR, the mass demonstrated slightly prolonged T1 and T2 signals (slightly hypointense on T1WI and slightly hyperintense on T2WI relative to myocardium) and heterogeneous enhancement after contrast administration (2); critically, there was no evidence of invasive growth or malignant features on either TTE or CMR, and immunohistochemical staining confirmed the positivity of the mass for endothelial markers (CD34 and CD31) (1, 22). These findings collectively ruled out the differential diagnoses (atrial myxoma, cardiac lipoma, metastatic tumors) and confirmed the benign, angiomatous nature of the lesion.

### 3.3 Value of multimodal imaging

This case highlights the complementary role of TTE and CMR in the diagnosis and management of right atrial hemangiomas. TTE rapidly identified the mass and evaluated its functional impact, specifically the partial tricuspid obstruction caused by the lesion. This information was critical for determining the urgency and necessity of surgical intervention. CMR clarified the anatomical origin of the mass, which was attached to the right atrial posterior wall and interatrial septum, and provided detailed tissue characterization to support its benign vascular nature. Quantitative DWI/ADC analysis offers objective, quantifiable evidence for differentiating benign vascular lesions from malignant tumors. The ADC value of 1.58 × 10⁻^3^ mm^2^/s in this case aligns with the typical features of benign hemangiomas, and serves as a valuable supplement to conventional T1WI, T2WI and LGE sequences. These CMR findings were essential for excluding malignant tumors and formulating a safe, targeted surgical plan.

### 3.4 Limitations and future follow-up

This case report has two main limitations that should be noted. First, the causal relationship between the right atrial hemangioma and the patient's prior intracerebral hemorrhage was not definitively confirmed. On one hand, no embolic material originating from the cardiac hemangioma was identified on clinical evaluation, and the absence of an intracardiac shunt makes a paradoxical embolism mechanism etiologically unlikely. On the other hand, dedicated cerebrovascular imaging was not performed at the time of the hemorrhagic event, so a concurrent intracranial hemangioma as the primary source of bleeding cannot be definitively ruled out. Second, the current follow-up period is relatively short, and long-term follow-up data are currently lacking. Long-term regular follow-up has been planned, including TTE at 6 months and CMR at 12 months postoperatively, to confirm no tumor recurrence and evaluate long-term surgical outcomes.

## 4 Conclusion

The multimodal imaging workflow integrating TTE for rapid functional assessment and CMR for quantitative tissue characterization (including DWI/ADC analysis) is a powerful strategy for the accurate diagnosis and surgical planning of rare right atrial hemangiomas. TTE enables rapid initial detection of the mass and comprehensive assessment of its functional impact on the heart (such as valvular obstruction), while CMR provides superior anatomical localization of the mass and detailed characterization of its tissue composition (including vascularization and benign features). Accurate preoperative imaging based on these two modalities is essential for guiding safe surgical resection of right atrial hemangiomas, which in turn improves patient outcomes. Long-term follow-up of patients with right atrial hemangiomas is also essential to confirm the benign nature of these lesions and to rule out the possibility of recurrence.

## Data Availability

The original contributions presented in the study are included in the article/Supplementary Material, further inquiries can be directed to the corresponding author.

## References

[1] Munteanu IR Novaconi RC Merce AP Dima CN Falnita LS Manzur AR. Cardiac hemangiomas: a five-year systematic review of diagnosis, treatment, and outcomes. Cancers (Basel). (2025):17:1532. 10.3390/cancers17091532 40361457 PMC12071036

[2] Grigorescu AE Novaconi RC Munteanu IR Manzur AR Zus AS Lazar M-A. Cardiac hemangioma in the right atrium: diagnostic challenges, imaging clues, and a novel algorithm for differential diagnosis. Life. (2025):15:1816. 10.3390/life15121816 41465755 PMC12734070

[3] Tavernese A Cammalleri V Mollace R Antonelli G Piscione M Cocco N. The role of advanced cardiac imaging in monitoring cardiovascular complications in patients with extracardiac tumors: a descriptive review. J Cardiovasc Dev Dis. (2025):12:9. 10.3390/jcdd12010009 PMC11765722 39852287

[4] Zhao L Su X Song X Ding Y Wang Q. A massive cardiac lipoma in the right atrium with multimodality imaging. J Cardiothorac Surg. (2024) 19:646. 10.1186/s13019-024-03162-3 39702165 PMC11658371

[5] Adomat F Steffen DA Suter-Magpantay L Linka A Weber L. Case report: a non-invasive approach to diagnosis and management of pericardial haemangioma. Eur Heart J Case Rep. (2024) 8:ytae545. 10.1093/ehjcr/ytae545 39430675 PMC11489877

[6] Tang W Tang M Li Y Yang J. Surgical treatment of a giant cardiac hemangioma in right atrium with pulmonary vein compression. Eur J Med Res. (2025) 30:1130. 10.1186/s40001-025-03394-6 41250155 PMC12625646

[7] Tartarin H Morotti A Van Etten ES Hausman-Kedem M Charidimou A Jouvent E. Uncommon causes of nontraumatic intracerebral hemorrhage. Stroke. (2024):1416–27. 10.1161/STROKEAHA.123.043917 38572651

[8] Chen Y Tang W Huang X An Y Li J Yuan S. Mitophagy in intracerebral hemorrhage: a new target for therapeutic intervention. Neural Regen Res. (2023) 19:316–23. 10.4103/1673-5374.379019 PMC10503626 37488884

[9] Kent DM Wang AY. Patent foramen ovale and stroke: a review. JAMA. (2025) 334:1463-73. 10.1001/JAMA.2025.10946 40720119

[10] Ariyada K Yamagishi K Kihara T Muraki I Imano H Kokubo Y. Risk factors for intracerebral hemorrhage by five specific bleeding sites: japan public health center-based prospective study. Eur Stroke J. (2024) 10:23969873241290680. 10.1177/23969873241290680 PMC11556633 39417686

[11] Hilkens NA Casolla B Leung TW De Leeuw F. Stroke. Lancet (London, England). (2024) 403:2820–36. 10.1016/S0140-6736(24)00642-1 38759664

[12] Peng J Chen D Ma X He Y Xia J. Tracing the source: suspicious right atrial mass. Acta Cardiol. (2025) 80:945–6. 10.1080/00015385.2025.2476889 40059479

[13] Gomase S Kuthe S Sonkusale M. Case report-right atrial myxoma with total anomalous pulmonary venous connection in neonate. Int J Surg Case Rep. (2022) 97:107438. 10.1016/j.ijscr.2022.107438 35908453 PMC9403170

[14] Ramcharan P Katwaroo A Maharaj M Seecheran V Lalchansingh D Seecheran R. Giant right atrial myxoma presenting with right heart failure. J Investig Med High Impact Case Rep. (2025) 13: 23247096251329706. 10.1177/23247096251329706 40145594 PMC11948545

[15] Antoniak R Kompa M Burdach R Grabowska-Derlatka L Slowikowska A Rowinski O. An atypical radiographic appearance of a cardiac myxoma: case report and review of the literature. Folia Morphol (Warsz). (2023) 82: 391–5. 10.5603/FM.a2022.0040 35411543

[16] Aquaro GD De Gori C Faggioni L Parisella ML Cioni D Lencioni R. Diagnostic and prognostic role of late gadolinium enhancement in cardiomyopathies. Eur Heart J Suppl. (2023) 25:C130–6. 10.1093/eurheartjsupp/suad015 37125322 PMC10132607

[17] Ezeani M Noor A Donnelly PS Niego B Hagemeyer CE. Assessment of the epi-pericardial fibrotic substrate by collagen-targeted probes. Sci Rep. (2022) 12:5702. 10.1038/s41598-022-08688-x 35383230 PMC8983671

[18] Yan Y Ma C Sandeep B Su X Xiao Z. Surgical resection of a lipoma of the right atrium: a case report. Medicine (Baltimore). (2025) 104: e41848. 10.1097/MD.0000000000041848 40128076 PMC11936591

[19] Bajdechi M Onciul S Costache V Brici S Gurghean A. Right atrial lipoma: a case report and literature review. Exp Ther Med. (2022) 24: 697. 10.3892/etm.2022.11633 36277161 PMC9535399

[20] Ztati M Dahmane W Moha WA El Hattaoui M. Malignant right atrial mass with Inferior vena Cava extension presenting as Budd-Chiari syndrome: a multimodality imaging case report. Cureus. (2025) 17: e96345. 10.7759/cureus.96345 41367464 PMC12683076

[21] Hu Z Yuan S Mou Y. Multiple thrombi mimicking metastases in the right atrium of patients with non-Hodgkin's Lymphoma diagnosed by multimodal cardiac imaging: one case report. J Cardiothorac Surg. (2024) 19: 165. 10.1186/s13019-024-02650-w 38561816 PMC10985842

[22] Rahouma M Mohsen H Morsi M Khairallah S Azab L Abdelhemid M. Prevalence, diagnosis, and treatment of cardiac tumors. A narrative review. J Clin Med. (2025):14:3392. 10.3390/jcm14103392 40429390 PMC12111963

