# Supplementary material for: Case Report: A large right atrial hemangioma: multimodal imaging and postoperative follow-up
Source: Front Radiol. 2026 Jul 30;6:1821324. doi: 10.3389/fradi.2026.1821324 (PMC13467978; doi:10.3389/fradi.2026.1821324)
Supplement: Supplementary file 1 [file Image1.pdf]

Supplementary Figure

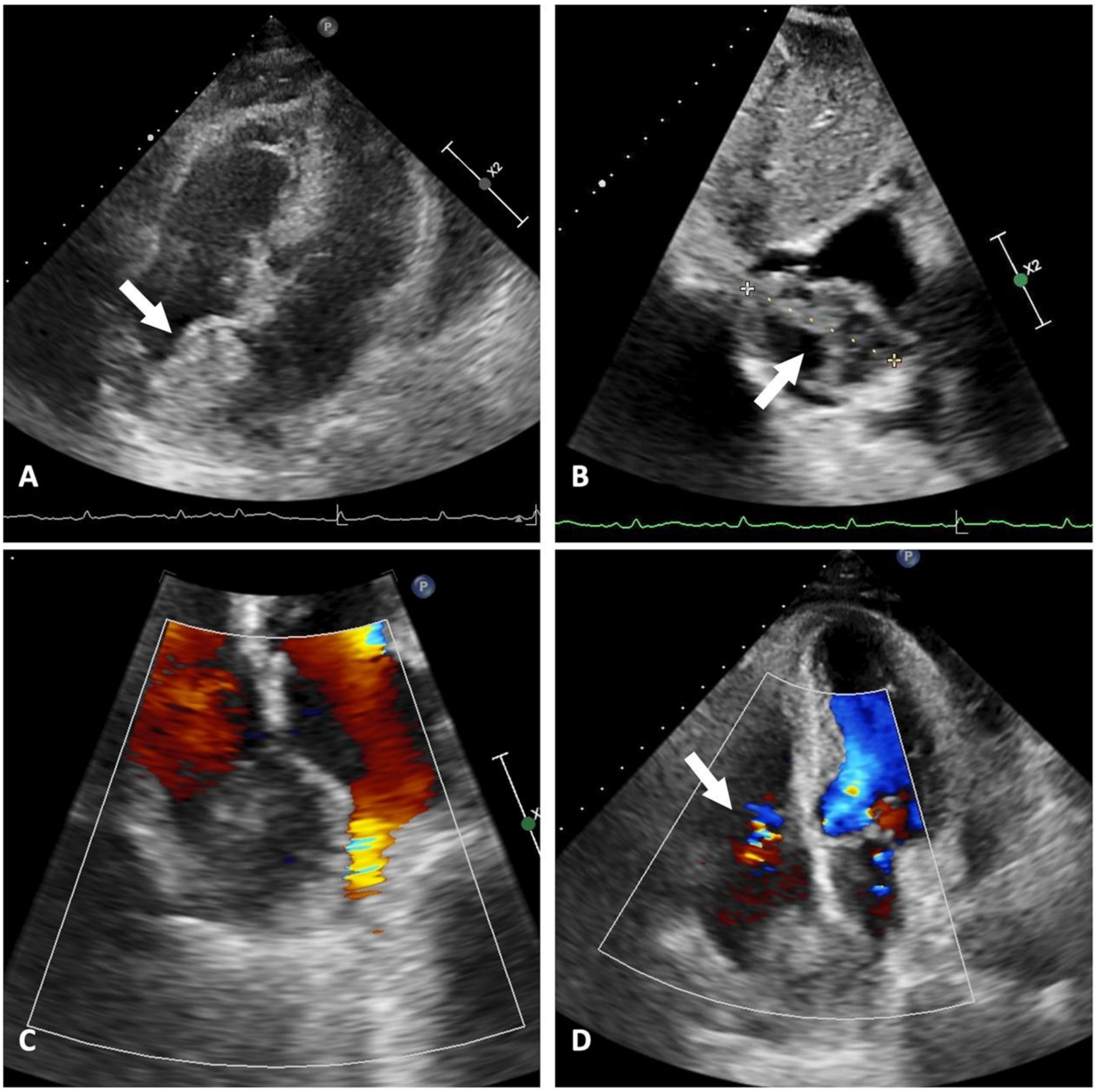

Supplementary Figure. Transthoracic echocardiographic findings at 2 weeks after surgical resection.

Panels A and B: Two-dimensional echocardiography shows a slightly hypoechoic lesion measuring 6.5×4.9×4.8 cm in the right atrium (white arrows). The lesion attaches to the mid-upper interatrial septum via a broad base with ill-demarcated borders, presenting an irregular contour and heterogeneous echotexture with small patchy hypoechoic to anechoic areas inside. No obvious mobility is observed during the cardiac cycle. The superior margin of the lesion is adjacent to the superior vena cava orifice, the inferior margin is close to the inferior vena cava orifice, and the right pulmonary vein is compressed. Panel C: No blood flow signal was detected within the lesion. Panel D: Color Doppler flow imaging demonstrates a mild systolic regurgitant jet on the right atrial side of the tricuspid orifice, consistent with mild tricuspid regurgitation (white arrow).
